# Grave Injustices to Medical Men

**Published:** 1919-02-08

**Authors:** 


### THE EDITOR'S LETTER-BOX.

**[Correspondence on all subjects is invited, but we cannot in any way be responsible for the opinions expressed by our correspondents, who must give their name and address as a guarantee of good faith, but not necessarily for publication. Correspondents are reminded that brevity of style and conciseness of statement greatly facilitate early insertion.]**

### Grave Injustices to Medical Men.

*To the Editor of* The Hospital.

Sir,—With reference to the article under the above title in The Hospital of February 1, I quite agree we certainly have not received much recognition from a so-called grateful country. How many men sunk their all in the original purchase of their practice, and either left it to join the Army, or now find practically nothing to return to? Is the country going to give or even lend money in sufficient quantity to those men to buy themselves a new practice? No—in fact, surely the bulk of those men—namely, the temporary commissioned men—are not to even get the War Gratuity or Bonus (of so many days' pay) that is given to other officers. What is the value of a £60 a year bonus to those medical men—in most instances it has already gone to pay expenses; and how far would even another £60 go towards buying a practice? Medical men do not publish abroad these facts as a rule, they suffer in silence, but surely now is the time to speak up and let the general public know. The average man wants the best part of £1,000 to buy a practice that will keep him and his wife and family in a reasonable manner, considering the position that he is expected to keep up. Why should not the country enquire into each medical man's case, and, according to his means that he has available, advance him the rest of the money that is necessary to place him in a reasonably average position. Surely this could be done in some form of a loan on the practice, the Government acting as security or surety; or possibly each man might be provided with a life insurance policy, on which he could raise the money with a bank, the Government still acting as guarantor, and the man on his part could pay back so much each year from his practice, clearing the whole say in six or ten years. Such a scheme would not be any excess of generosity, but it would certainly enable many of us who have given our all to live.

Yours truly,

A Sufferer.

